# Raising Capacity to Address Mental Health Concerns in Costa Rica

**DOI:** 10.7759/cureus.32481

**Published:** 2022-12-13

**Authors:** Thomas A O'Mara, Kurt Angstman, Douglas B Spicer, Enid Campos, Natalie C Erbs, Joseph W Furst, Mark D Williams

**Affiliations:** 1 College of Osteopathic Medicine, University of New England, Biddeford, USA; 2 Family Medicine, Mayo Clinic, Rochester, USA; 3 Psychiatry, The Center Clinic, Rochester, USA; 4 Psychiatry and Psychology, Mayo Clinic, Rochester, USA

**Keywords:** suicide risk, medical trips, medical education, survey analysis, interprofessional education and collaboration, global health education, suicide prevention, bipolar, mental health and suicide, underserved population

## Abstract

Background

A family medicine team based out of Mayo Clinic, Rochester assembled in 2019 to provide home visits and direct care to underserved populations of patients in La Cruz, Costa Rica. In addition to the provision of direct patient care, our team was interested in conducting a community needs-based assessment to identify an area for provider education efforts and the local providers on a chronic health issue using local feedback and physician data. Suicide awareness and prevention were identified as a priority based on rising suicide rates as well as limited psychiatry services in the area, with some major providences having ~0.60 psychiatrists available per 100,000 people. Our group provided a half-day educational course on mental health topics related to suicide awareness for local health workers. The primary objective of this study was to evaluate any lasting changes in practice, confidence, and knowledge among local health workers attributable to our training and add to the limited research on this topic.

Methods

Two groups of participants (81) from local hospitals were recruited via local providers and divided into two morning and afternoon groups on a single day. Each group comprised primary care providers, nurses, social workers, and finance officers. Both were given the same educational presentation that could be broadly applied to each various role. Our team provided lectures on mental health, as well as how to improve personal resilience. Locally medically trained translators were used. Pre and post-lecture surveys gathered demographic data, experience with these mental health issues, and confidence in addressing mental health concerns. Pre and post-lecture surveys, including open-ended as well as Likert scale and multiple-choice questions, were handed out at the beginning and end of each lecture to all participants. A four to six months follow-up survey was delivered by email using SurveyMonkey to evaluate retention and impact of educational materials.

Results

The initial two groups of participants (n = 81) were aged 23-60 years (mean: 43), and 67% (39) were female. Work experience ranged from 0 to 37 years (mean: 14) with 64% (37) doing direct patient care. Preliminary lecture content data from participants (n = 44) demonstrated an overall increase in correct responses by +15.4% from the pre-test (percent correct, 38.1%) to post-test (53.5%, p < 0.01). Individuals (n = 55) with past exposure to suicide were much more likely to report asking patients about suicide than those with no prior exposure (56.3% vs. 8.3%; p < 0.01). At the six-month follow-up with participants (n = 11), when asked about their confidence in learning objectives from the lecture given prior, the rates of low confidence decreased as well as the level of high confidence improved but was not statistically significant. The rate of low confidence of respondents' confidence in asking about mental health concerns decreased from 35.2% to 0% (p < 0.01).

Conclusions

Our group was able to successfully deliver lectures to a mixed audience of health workers in a region self-identified as struggling with mental health issues in Costa Rica. The surveys suggested learning occurred. A trend suggestive that the educational content improved the participants' confidence and knowledge components over time was noted. Future service trips may be able to build on this initial experience to improve on ways to raise capacity while delivering direct care to regions in need.

## Introduction

Global health service trips are provided by many organizations and individuals each year. Prior to the coronavirus disease 2019 (COVID-19) pandemic, recent studies have shown that the number of service trips was increasing every year [1], but there is limited research exploring the long-term impacts of the care or service given [2-4]. Common critiques of these service trips include their limited efficacy in sustaining change once the group has left, as well as inappropriate care stemming from cultural unawareness or assumptions being made [5-8].

While direct patient care during a service trip provides an immediate clinical impact on the patients cared for [4], limited research was found regarding raising the capacity of local providers to address chronic health issues. Recent studies have hypothesized that a greater impact is made if the care provided revolves around the needs of the community [5], along with providing education to promote long-term education to patients as well as local providers [9,10]. In addition to education, exploring the perceptions of the patient’s needs within the local community also provides a greater insight to address specific healthcare needs for future trips to the same area [11].

This study was performed concurrently with a planned health service trip to La Cruz, Costa Rica in 2019. A Mayo Clinic-affiliated medical team arranged to provide home visits and direct care to underserved populations of patients in La Cruz through the assistance of Vida Volunteer, a nonprofit volunteer group with links to local health services. La Cruz is a border county in the province of Guanacaste in northwestern Costa Rica. As largely a rural community and Guanacaste’s largest city, La Cruz is less than 20 km south of the border with Nicaragua. Tourism services have overcome agricultural activities as the main source of income for its current estimated population of 28,071 projected for 2022, which still faces several significant socioeconomic challenges [12]. Approximately 20% of its population corresponds to immigrants, mostly from Nicaragua, who are arriving to some socioeconomically challenged areas with limited health care [13]. Specifically in La Cruz, local providers expressed to our group how most of their mental health services are provided by general physicians, thus highlighting the reduced presence of specialized mental health care. Any other additional psychiatry services are only available in the hospital located more than four hours away in San Jose, the capital of Costa Rica. Because of this sparsity of resources, local providers were asking to receive mental health education during our visit.

As such, in addition to providing patient care and in consultation with the local medical community, interprofessional education (IPE) lectures covering mental health topics were constructed before our trip and provided to local healthcare employees in a single day. Each lecture covers topics designed to educate broadly and raise the local capacity of addressing mental health concerns for both providers and other healthcare employees.

The primary objective of this study was to evaluate if there were any lasting changes in practice, confidence, and knowledge among local health workers attributable to our training.

This article was previously presented as a meeting abstract at the Mayo Clinic Family Medicine Series in Rochester, MN in November 2020.

## Materials and methods

The Mayo-affiliated medical team consisted of six physicians, including two psychiatrists, two nurses, an emergency medical technician (EMT), as well as various undergraduate and medical students. Prior to embarking, we sought input from local health leaders in La Cruz on topics of interest to address chronic health issues and to provide specific care to the needs of the community. The needs of the community were assessed by asking the local health director and relying on Vida Volunteer staff to inform us directly. Post consultation, suicide awareness and prevention were identified as a priority. Local physicians presented local unpublished data showing rising suicide rates in that area, and they hypothesized this may be related to an influx of Nicaraguan refugees in the prior year, who are arriving from socioeconomically disadvantaged sites and traveling long distances without proper health care [13]. Our group agreed to offer a half-day course on mental health topics related to suicide awareness and prevention for interested local health providers in that region.

Two identical educational lecture series were given to two different audiences (am and pm groups) of local medical staff members working in La Cruz. Each group comprised primary care providers, nurses, social workers, and finance officers. Both were given the same educational presentation that could be broadly applied to each various role. The lecture series topics comprised depression, bipolar affective disorder, suicide awareness, suicide prevention, as well as how to improve personal resilience in both patients and participants themselves. For English-speaking lecturers from our group, English-to-Spanish translation was provided concurrently by a local official translator. Each lecture series was three hours long. Educational topics discussed were depression, bipolar affective disorder, suicide awareness and prevention, and how to improve resilience in both patients and healthcare providers. Each lecture was designed to cover a small range of topics tailored to the issues of the local area.

Participants attended the morning or afternoon lectures. The first two lectures on depression and bipolar disorder were given in Spanish by a psychiatrist from the Mayo Clinic team who was originally from Costa Rica. The second two lectures on suicide awareness and resiliency were given in English by Mayo Clinic physicians with a simultaneous translator who was a Costa Rican physician who had the materials in advance of the presentations.

Pre and post-lecture surveys comprising open-ended questions, as well as Likert scale and multiple-choice questions, were handed out at the beginning and end of each lecture to all participants (Appendix). These surveys gathered demographic information, experience with mental health issues, and confidence in addressing mental health concerns. They also assessed content questions based on the lecture used to assess pre and post-lecture content knowledge. Because not all participants were directly seeing patients, questions were asked about both personal and professional experiences with suicide or mental health issues. Surveys were translated to Spanish by one of our Vida Volunteer hosts, and the translations were checked for accuracy by two bilingual Mayo Clinic staff. These staff also translated survey responses that were in Spanish back to English.

A four to six months follow-up survey was delivered by email using SurveyMonkey® (https://www.surveymonkey.com/) to evaluate the participants’ retention of educational materials as well as confidence changes using the same questions from the initial survey (Appendix). In addition, we asked participants open-ended questions such as if there was any change in their practice, and if so, were there any new barriers they faced.

The data were entered into an Excel database (Microsoft Corporation, Redmond, WA). Statistical analysis was performed utilizing MedCalc Statistical Software (version 19.2.6; MedCalc Software Ltd, Ostend, Belgium; https://www.medcalc.org). Categorical testing was performed utilizing chi-squared or Fisher’s exact test (depending on the size of the groups tested) and continuous variables were evaluated via independent t-tests. A p-value of <0.05 was considered statistically significant. This study was reviewed by the Mayo Clinic Institutional Review Board and approved as an exempt study.

## Results

Lectures were delivered to 81 medical providers, and 58 filled out the survey; for a survey response of 71.6%. There were some surveys that were partially completed with various skipped questions; however, these surveys were included in the final analysis, as the questions answered were 100% complete. The respondents represented various backgrounds, including primary care providers, nurses, mental health providers, pharmacists, and finance/administration officers. Work experience ranged from 0 to 37 years (x̄ = 14) with 64% (37) involved in direct patient care. Regarding all participants who answered the following survey question (53), 37.7% reported having personally or professionally dealt with suicide, while the remaining 62.3% have not had experience at all. On a follow-up question regarding past exposure to any mental health exposure (48), 19% admitted to endorsed personal experience, 43.7% as professional, 29.2% as both professional and personal, and only 8.3% were unexposed.

The baseline confidence of all participants was surveyed using a Likert scale, as seen in Table 1. Almost half (42%) of the respondents answered they were highly confident that they would screen a patient for depression (n = 50). However, less than a quarter had high confidence in recognizing depression (21.2%) or a suicidal patient (24.6%). The confidence to recognize a bipolar patient was the lowest, with only 17% of respondents categorizing themselves as highly confident.

**Table 1:** Baseline mental health clinical confidence in Costa Rican participants in educational presentations by variable

N = 58		
Confidence in recognizing depression (range: 1-6) (n = 52)	Mean	3.7 (3.34 to 4.04)
	Median	4.0 (3.47 to 4.00)
	Low confidence (1-2)	15.4% (14)
	Moderate confidence (3-4)	63.5% (33)
	High confidence (5-6)	21.2% (11)
Confidence in recognizing bipolar disorder (range: 1-6) (n = 53)	Mean	3.4 (3.04 to 3.76)
	Median	4.0 (3.0 to 4.0)
	Low confidence (1-2)	24.5% (13)
	Moderate confidence (3-4)	58.5% (31)
	High confidence (5-6)	17.0% (9)
Confidence in recognizing suicidal patients (range: 1-6) (n = 53)	Mean	3.2 (2.79 to 3.62)
	Median	3.0 (3.0 to 4.0)
	Low confidence (1-2)	34.0% (18)
	Moderate confidence (3-4)	41.6% (22)
	High confidence (5-6)	24.6% (14)
Confidence in asking about mental health concerns (range: 1-6) (n = 54)	Mean	3.4 (2.86 to 3.84)
	Median	4.0 (3.00 to 4.67)
	Low confidence (1-2)	35.2% (19)
	Moderate confidence (3-4)	27.8% (15)
	High confidence (5-6)	37.1% (20)
Confidence in helping someone with mental health problems (range: 1-6) (n = 55)	Mean	3.6 (3.22 to 4.02)
	Median	4.0 (3.00 to 4.00)
	Low confidence (1-2)	23.6% (13)
	Moderate confidence (3-4)	52.8% (29)
	High confidence (5-6)	23.6% (13)
How likely are you to screen a patient for depression? (Range: 1-6) (n = 50)	Mean	3.8 (3.33 to 4.27)
	Median	4.0 (3.00 to 5.00)
	Low confidence (1-2)	24.0% (12)
	Moderate confidence (3-4)	34% (17)
	High confidence (5-6)	42% (21)

Forty-four participants completed a pre and post-test survey. The average score in the pre-test was 38.1%, increasing to 53.5% in the post-test, improving from a pre-lecture average score of 38.1% to a post-lecture average score of 53.5% (p < 0.01), as seen in Table 2. The degree of improvement in correct answers for lectures delivered in Spanish was not statistically different than those delivered in English. Pre to post-test scores improved for questions regarding a true/false question on suicide risk factors in patients (66.7% vs. 83.3%), differentiating suicide rates between bipolar vs. unipolar depression (0% vs. 18.2%), and the components of resiliency (52.6% vs. 68.4%), all showing significant improvement in scores (each with a p < 0.01). Positive trends were seen in the other five tested responses.

**Table 2:** Pre- and post-test questions with results of the entire group per question (% with correct answers to questions)

% Answers correct	Pre-test	Post-test	p
Q1. Major depression vs. adjustment disorder (n = 44)	43.2% (19)	56.8% (25)	0.46
				Q2. Diagnosis of depression criteria (n = 42)	50.0% (21)	61.9% (26)	0.06
Q3. Higher rate of suicide - bipolar vs. major depression (n = 44)	0.0% (0)	18.2% (8)	<0.01
Q4. Bipolar screening questions (n = 43)	30.2% (13)	41.9% (18)	0.09
Q5. Asking about suicide increases risk (n = 42)	66.7% (28)	83.3% (35)	<0.01
Q6. Suicide risk factors (n = 44)	40.9% (18)	54.5% (24)	0.05
Q7. Develop mental health issues from trauma (n = 36)	22.2% (8)	44.4% (16)	0.05
Q8. Component of resiliency (n = 38)	52.6% (20)	68.4% (26)	<0.01
				Total % answers correct	38.1% (127)	53.5% (178)	<0.01

The six-month follow-up survey was conducted using 29 email addresses from the pre and post-surveys and was used to communicate to participants who recorded their emails. Eleven participants responded (38% response rate). While the rates of low confidence decreased and the high confidence improved between the initial educational conference and six months later (Table 3), the numbers were not statistically significant, except for the respondents’ confidence in asking about mental health concerns (rate of low confidence decreased from 35.2% to 0%).

**Table 3:** Six-month follow-up survey responses for participants in Costa Rica by the level of confidence

n = 11	Baseline	Follow-up	p
Changes in practice since interprofessional education (IPE)?	N/A	100% yes	
Levels of high confidence (Likert 5 or 6)			
Recognizing bipolar depression	17.0%	36.4%	>0.05
Recognizing a suicidal patient	24.6%	27.3%	>0.05
Asking about mental health concerns	37.1%	25.0%	>0.05
Helping someone with mental health concerns	23.6%	27.3%	>0.05
Screening a patient for depression	42.0%	54.5%	>0.05
Levels of low confidence (Likert 1 or 2)			
Recognizing bipolar depression	24.5%	9.1%	>0.05
Recognizing a suicidal patient	34.0%	0.0%	>0.05
Asking about mental health concerns	35.2%	0.0%	<0.05
Helping someone with mental health concerns	23.6%	10.0%	>0.05
Screening a patient for depression	24.0%	9.1%	>0.05

In our follow-up survey, open-ended questions were given to help further develop an insight into the needs of the local population. When asked if their practice has changed since they participated in our IPE, 100% of the participants responded with “yes” (Table 3). While this is highly subjective and attributive to social pressures, it is worth noting for future studies to follow changes in suicide ideation and depression diagnosis in a six-month follow-up after surveys. When given the option to elaborate on such changes, we found healthcare providers were more aware of changes in “patient personality, expression, and behavior at appointments.”

Some other feedback was they were able to “deliver more personalized assistance” to patients and were “more curious” about learning the intricacies of psychiatric illnesses and looking into the topic rather than individual parts. Follow-up for the elaboration of these subjective responses was not conducted.

Regarding the resilience lecture, many providers reported they were able to utilize components of the resilience seminar for themselves along with teaching the foundation of good health practice to patients. One physician commented (translated from Spanish), “I better consider the importance of sleep hygiene, doing exercise more frequently... I learned that I must control myself to manage the term resilience.”

When asked to describe a “major obstacle a provider must overcome to improve the care of patients with mental health problems,” we found “stigma” was mentioned two times, “family abandonment patients into health system” and “problems finding sufficient mental health resources” were each mentioned two times each, along with a mention of “delays in care” and “lack of healthcare technology.” Future research may want to focus on these broad topics and attempt to identify these obstacles prior to medical trips.

## Discussion

Results are suggestive that there seems to be a trend in improved confidence in healthcare providers through our IPE lectures, with data, although significantly limited by response rate, showing long-term confidence regarding asking patients about mental health concerns. The ability of providers to recognize bipolar disorder, depression, and suicidal patients showed improvement via responses. Teaching to a group of providers and local healthcare workers seems to lead to sustainable educational impacts and improves their overall confidence in areas that they were previously not as confident in; however, a far greater number of respondents should have been recruited to improve the power of our study. As seen in previous studies [5], through the identification of obstacles prior to our trip with communication through the local providers collaborated with, we believe our IPE was more effective and able to target a lecture based on a community need.

By utilizing a multiple-choice style quiz on the lecture topics given, we were able to know if participants were able to understand our lectures as well as determine if learning had occurred. There was an increase of +15.4% in percent correct answers on the post-test vs. the pre-test, indicating participants were able to understand our translated content and accurately answer topic-related questions, but also suggests that some learning of information occurred. This observation is limited severely by the immediacy of the post-lecture survey, which was given right after the lecture ended. While limited, we observed a mild increase in percentage correct on the post-test in the following three areas: (1) suicide risk factors in patients (66.7% vs. 83.3%), (2) differentiating between suicide rates in bipolar vs. unipolar depression (0% vs. 18.2%), and (3) the components of resiliency (52.6% vs. 68.4%), with p values < 0.01, as seen in Table 2. The other five content-related questions exhibited an overall improvement of answers correct, but without statistical significance. Overall, the trend shows the acquisition of knowledge but is limited by the immediacy of the survey. Future suggestions are to deliver the post-lecture survey at a later time, as this may have made our data more convincing.

Our research adds to the paucity of short-term mission trips research by exploring not only the knowledge gained, similar to previous studies [9,10], but by exploring specific changes in the confidence of participants. Through our comparison of baseline confidence levels to the follow-up survey six months later, we were able to see the lasting positive impact of our IPE. Given the small number of participants, these observations are simply trends. Compared to the baseline survey, there was increased confidence seen in “asking patients about mental health concerns.” The number of participants indicating low confidence decreased from 35% to 0% (p < 0.05), suggesting they may be more aware to ask about mental health concerns after the IPE was delivered. However, high confidence levels in this category also dropped (37.1% to 25%). While this latter finding indicates decreased levels of high confidence, it is possible that individuals who initially were highly confident in that arena realized post-IPE that their confidence to ask was not as high as they originally thought. While not statistically significant, the other five categories showed increased levels of confidence as well. The number of participants with low confidence levels dropped, while there was an increase in the number of participants marking high confidence. The average change in percentages was -19% in low confidence and +9.5% in high confidence within these five categories.

While it is important to look at the hard data gathered from the surveys, asking open-ended questions allowed us to find immediate barriers the community is facing as well as interest in future topics for IPE, as shown in previous research [11]. The needs of the community were assessed by asking the local health director and relying on Vida Volunteer staff to inform us directly. Similarly to previous studies addressing the long-term impacts of these types of trips [10], by identifying barriers from local providers and medical staff, we are able to inform the health community of patient needs, and by exploring other interests, we are able to potentially compound the benefits of continuing education through follow-up visits. One of the largest barriers participants noted was the lack of mental health resources in their area, as one participant expressed the “need for a psychologist or mental health nurse” and another stated the “need for a specialized center with capable personnel to resolve these (mental health) situations.” Regarding future topics, many participants expressed interest in “adolescent mental health” education and how to “approach substance use.” There was also a request for a more “in-depth lecture series.” Going forward, while providers may understand what they need from a medical standpoint, delivery of a pre-trip instrument for data collection from the community members itself could prove to be valuable information in addition to the immediate medical needs, as seen from local providers.

One of the biggest criticisms of service trips has been their short duration with limited research into any sustainable change or benefit to the community once a group has left [2-4,6-8]. This group was piloting a model that not only provided short-term relief through direct patient care but also sought to bring about sustainable change by educating local health providers on a topic of their choice. By identifying local needs prior to the trip, we believe we were able to make a greater impact by revolving care around the needs of the community and promoting long-term and sustainable change by educating local providers. Our use of pre and post-surveys allowed us to assess the efficacy of the IPE, as well as identify potential topics for IPE on future service trips. We were able to successfully assess content acquisition and retention, long-term confidence levels in these topics, and the immediate barriers of the community. The long-term impact of our IPE can perhaps best be judged by the answers to the survey given six months after the trip where 100% of respondents indicated that they had changed their practice since participating in the IPE.

Through our educational lecture series combined with survey collection analysis, a trend was noted suggestive that the preplanned educational content improved the participants’ confidence as well as knowledge in lecture components over a six-month period, particularly in addressing mental health and suicide concerns, along with further identifying the other areas of need as described from our subjective findings. While some of the questions did not reach statistical significance, we feel confident the positive trends toward improving overall confidence levels of individuals regarding mental health topics were significant enough to report. It was evident that an educational lecture series on a specific topic improved the participants’ confidence and knowledge components over time and most importantly showed a sustainable change in their practice. Through this work, we believe we have successfully improved the quality of care of local providers and certainly laid the foundation for future trips to build upon.

Lessons learned

For those who wish to embark on this local area, we suggest acquiring a bilingual staff (if possible) and surveying topics of interest beforehand to ensure long-term impact.

If using a survey that will eventually have a follow-up, we would highly suggest utilizing a survey that can be distributed using a link via email or text message. Instead of trying to decipher each participant’s unique handwritten emails, by providing a text-based or email-based survey, we may have had increased numbers of responses. Furthermore, we believe having a computer or tablet available for participants to type their name and email would lead to a greater number of valid email addresses. We would also like to note that a lot of success in retention rates was because of the indoor environment in which we taught, and setting up suitable conditions beforehand was important to retain participant and lecturer attention span. With a recent increase in virtual learning, the long-term educational impact of these trips may be augmented with additional or follow-up educational lectures provided online for local providers as well.

Limitations

Some limitations to note were, in addition to creating a more effective survey, language and cultural barriers may have led to misinterpretation of the questions in our survey. We also acknowledge the small sample of participants and not all participants filling out the survey fully may have skewed results. The follow-up survey also only had very few participants as well. In doing these types of activities, commonly people drop off, limiting the conversations and amount of results we were able to obtain from the follow-up survey. While practices were noted to be changed in 100% of the participants who answered, the lack of objective information resulted in no way to verify changes.

## Conclusions

Our group was able to successfully deliver content lectures to a mixed audience of health workers in a region identified as struggling with mental health issues in Costa Rica. Pre and post-lecture surveys indicate that learning had occurred. A trend was noted that was suggestive that the preplanned educational content seem to have improved the participants’ confidence as well as knowledge in lecture components over a six-month period. Future service trips may be able to build on this initial experience to improve on ways to raise capacity while also delivering direct care, and it further shows that seeking the needs of the local community in which trips are being conducted may be most beneficial to achieving a long-lasting impact for short-term trips.

## Figures and Tables

**Figure 1:**
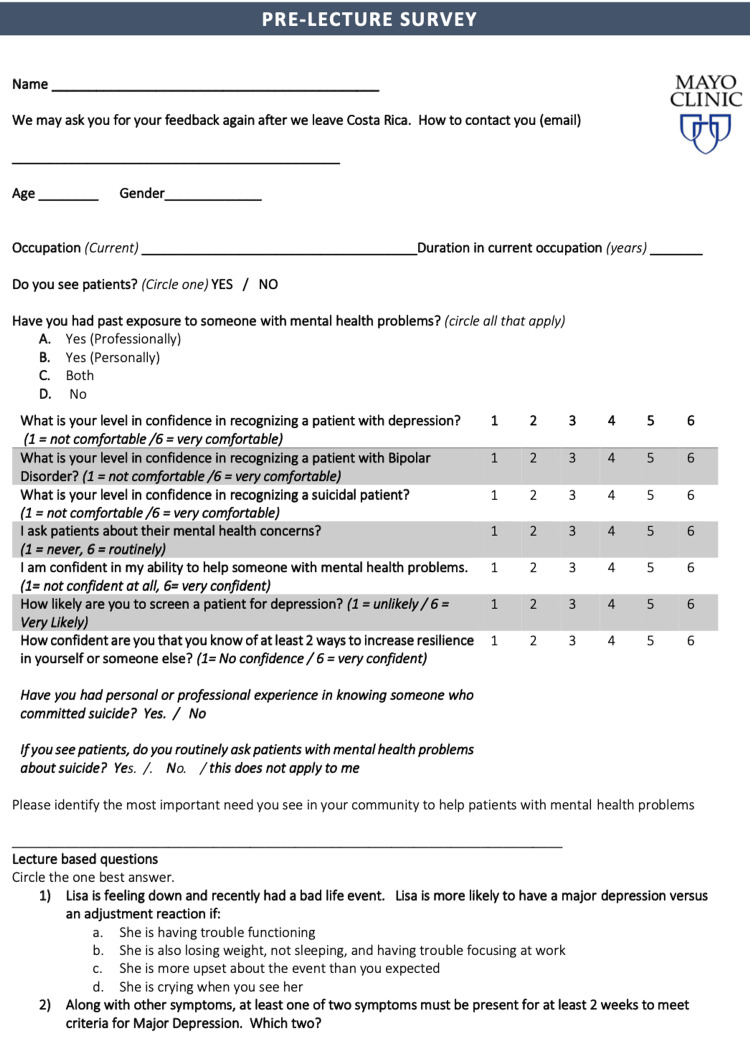
English pre-lecture survey (page 1)

**Figure 2:**
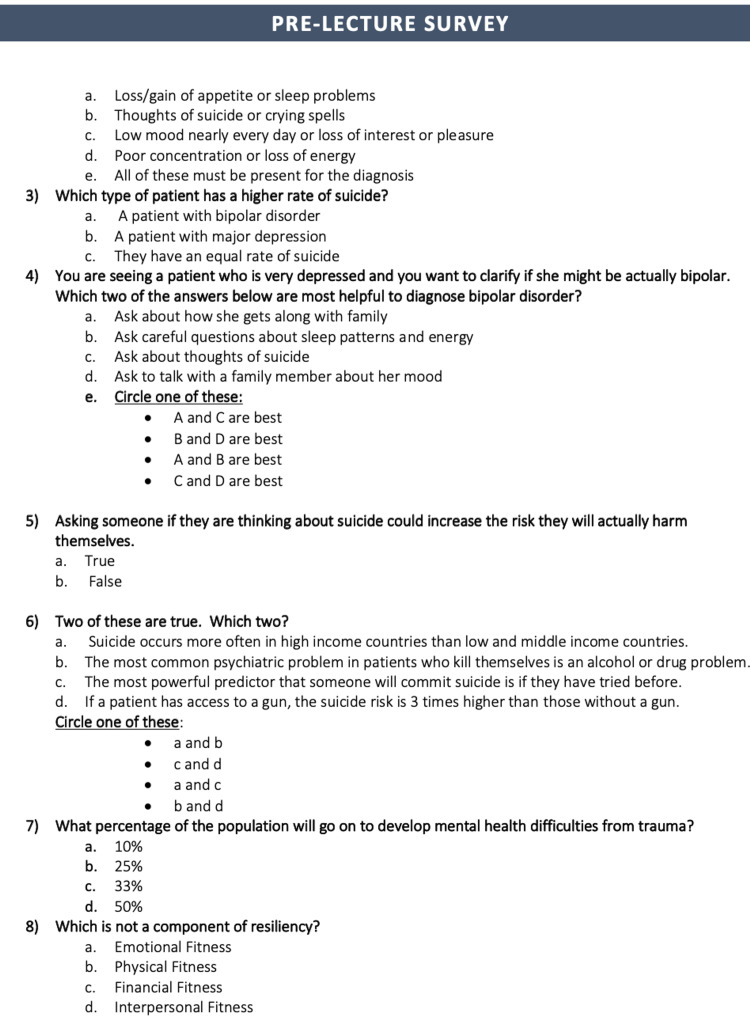
English pre-lecture survey (page 2)

**Figure 3:**
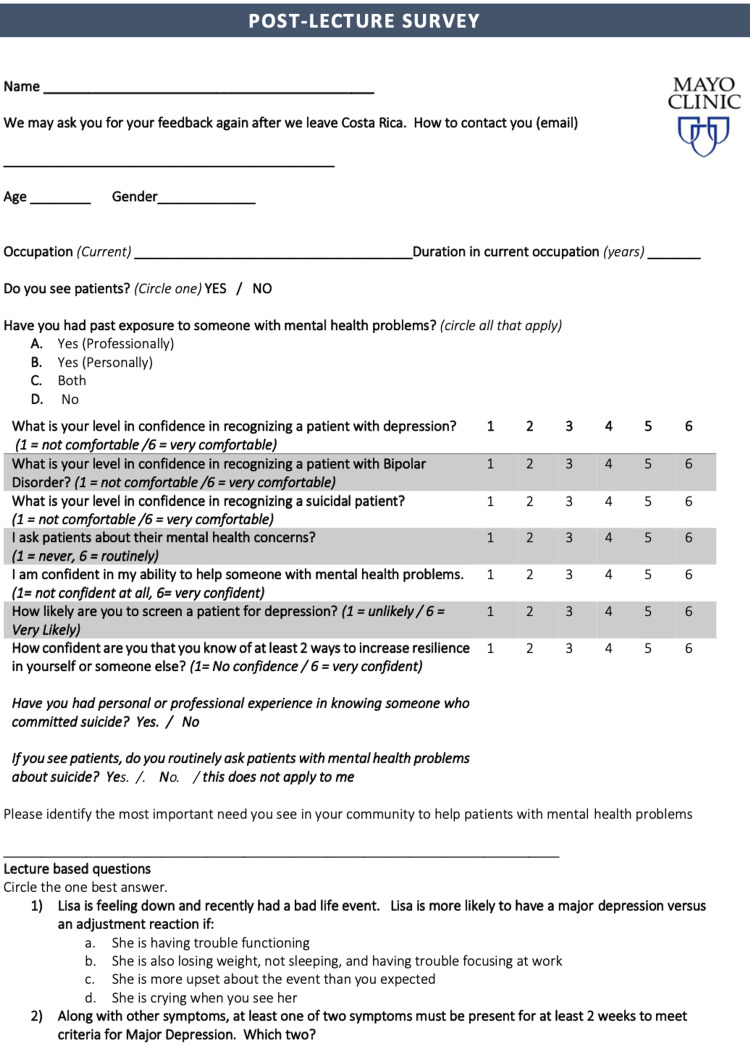
English post-lecture survey (page 1)

**Figure 4:**
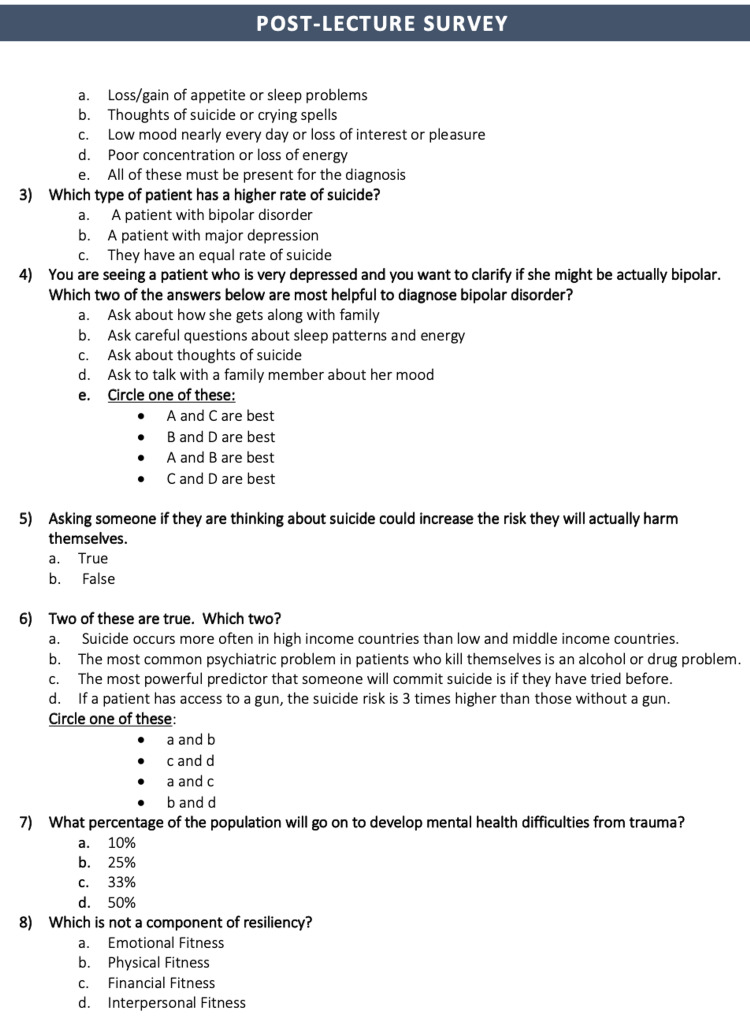
English post-lecture survey (page 2)

**Figure 5:**
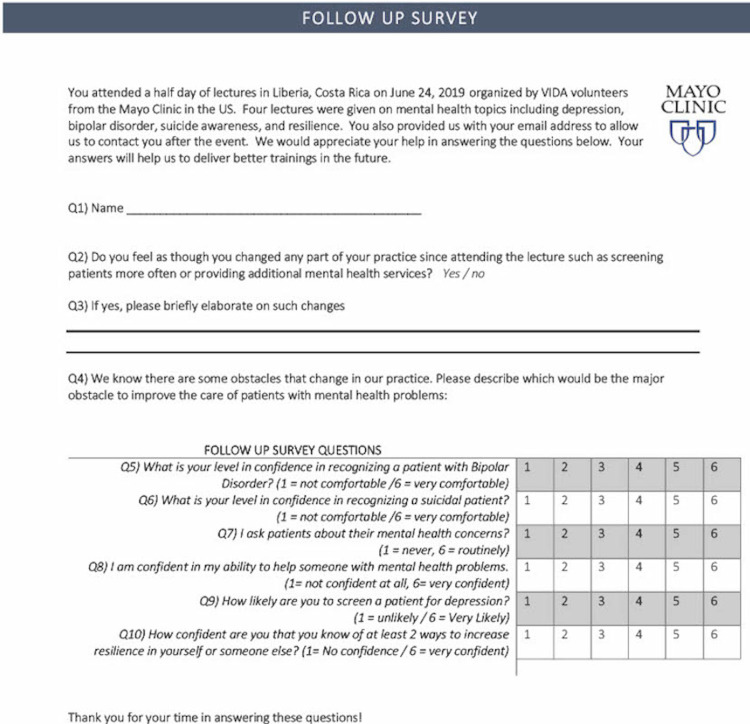
English follow-up survey

**Figure 6:**
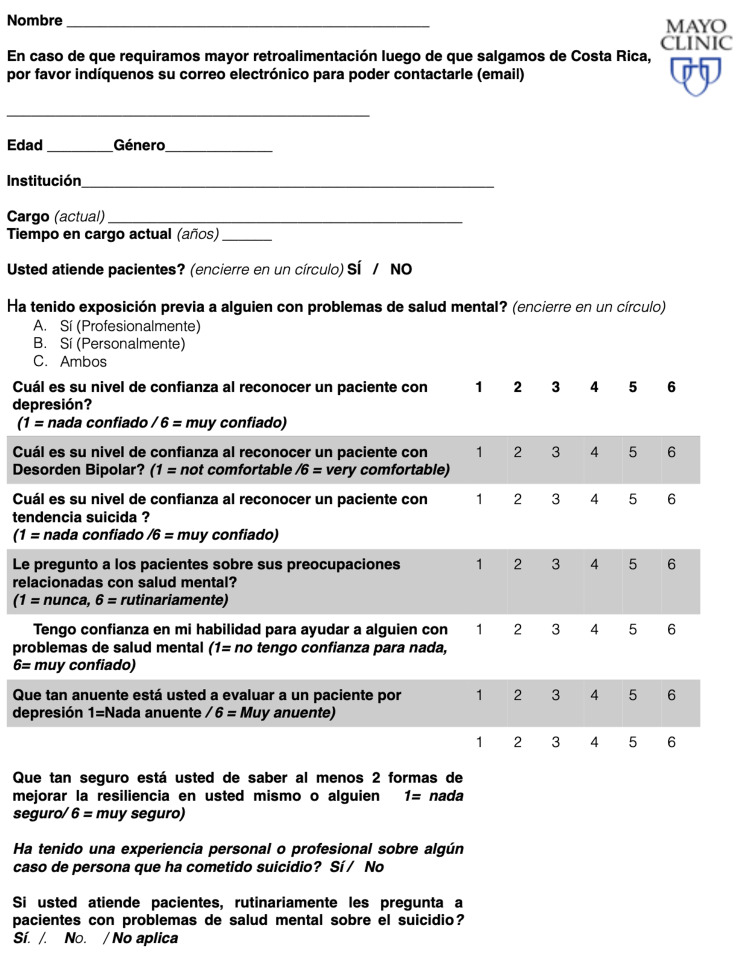
Spanish (translated) pre/post-lecture survey (page 1)

**Figure 7:**
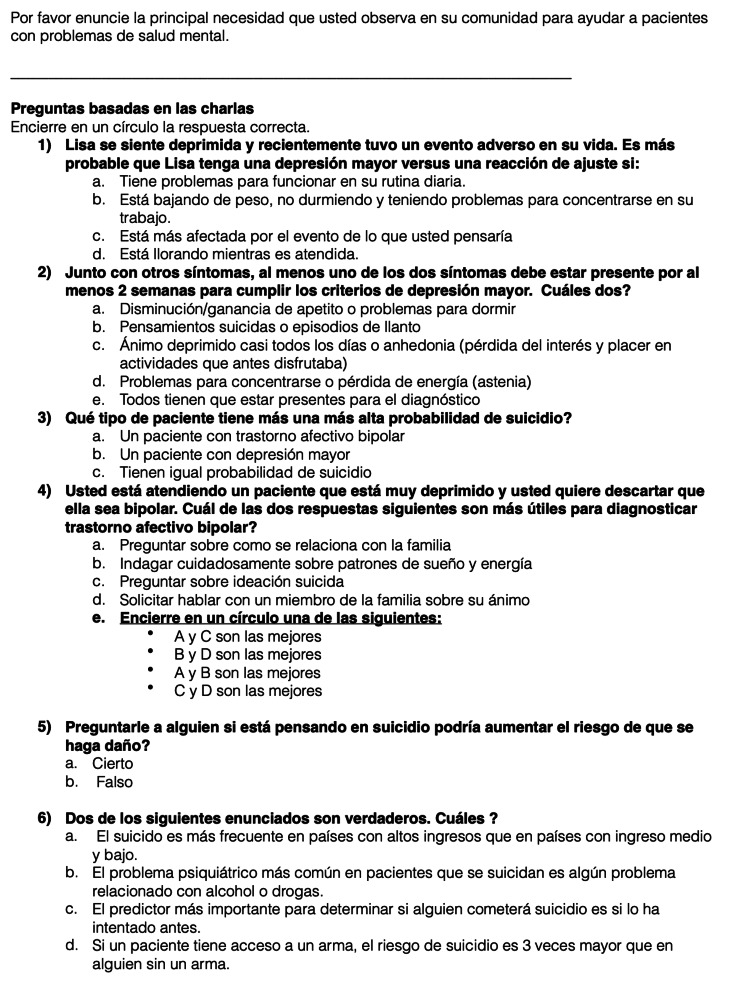
Spanish (translated) pre/post-lecture survey (page 2)

**Figure 8:**
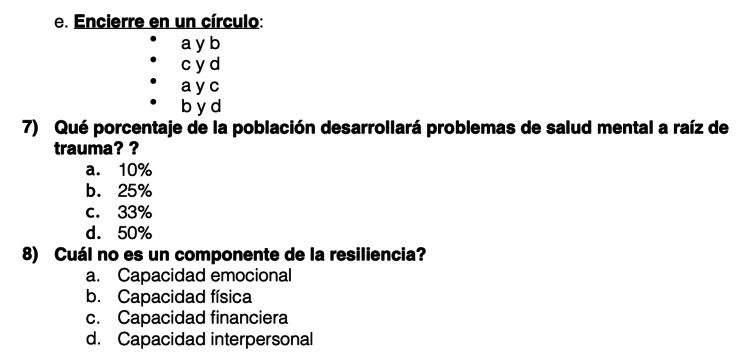
Spanish (translated) pre/post-lecture survey (page 3)

**Figure 9:**
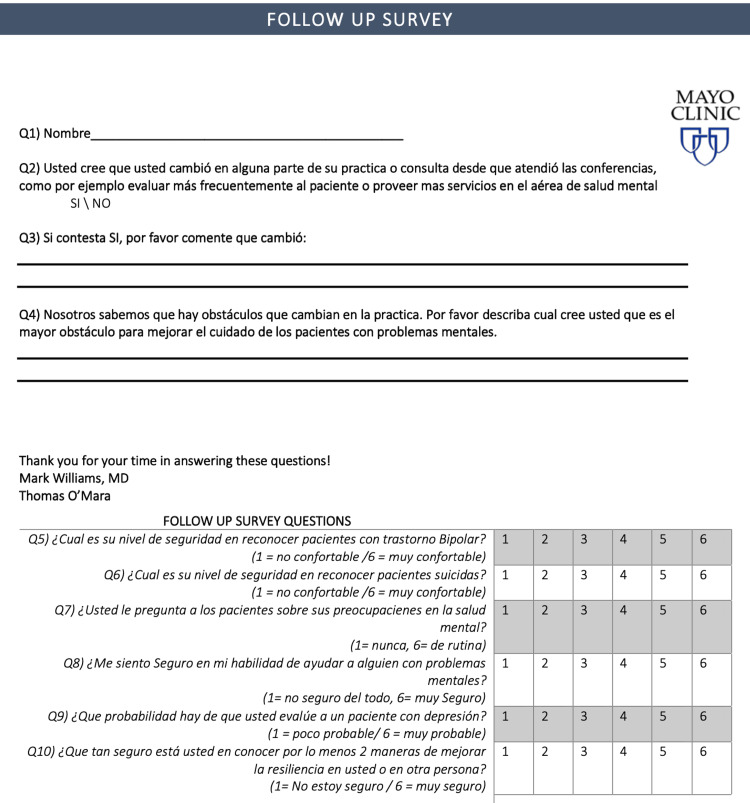
Spanish (translated) follow-up survey

**Figure 10:**
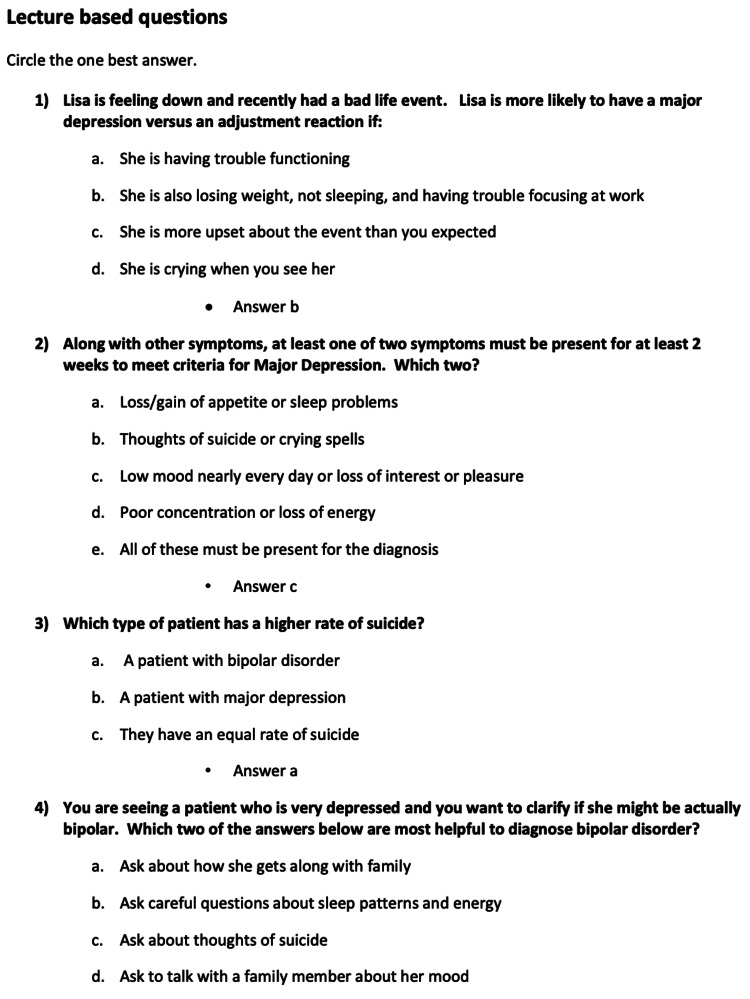
Answers to multiple choice questions on pre/post-lecture surveys (page 1)

**Figure 11:**
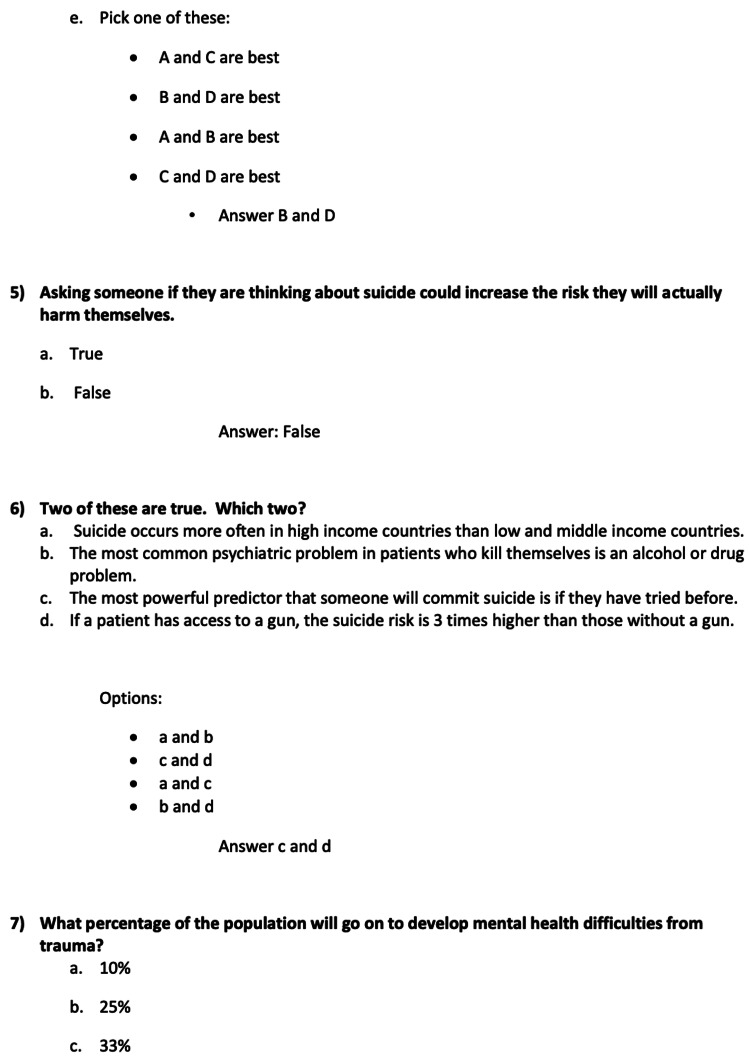
Answers to multiple choice questions on pre/post-lecture surveys (page 2)

**Figure 12:**
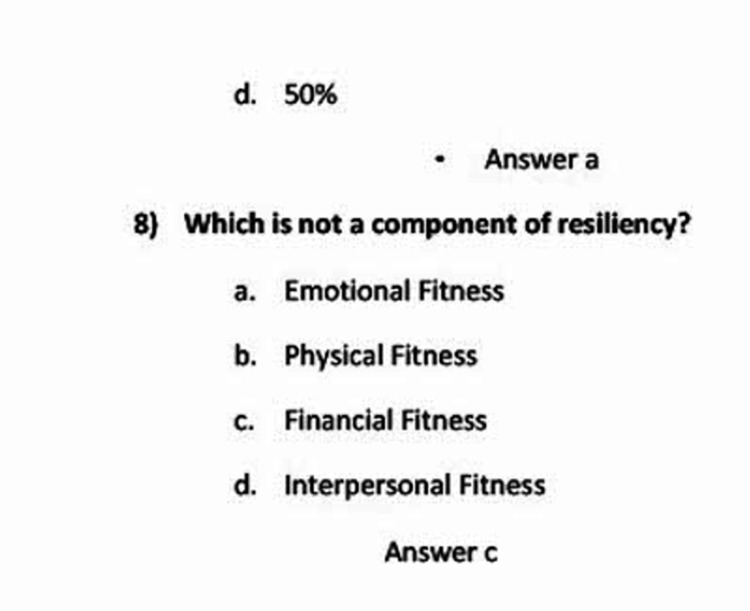
Answers to multiple choice questions on pre/post-lecture surveys (page 3)
